# Visual and quantitative evaluation of [^18^F]FES and [^18^F]FDHT PET in patients with metastatic breast cancer: an interobserver variability study

**DOI:** 10.1186/s13550-020-00627-z

**Published:** 2020-04-19

**Authors:** Lemonitsa H. Mammatas, Clasina M. Venema, Carolina P. Schröder, Henrica C. W. de Vet, Michel van Kruchten, Andor W. J. M. Glaudemans, Maqsood M. Yaqub, Henk M. W. Verheul, Epie Boven, Bert van der Vegt, Erik F. J. de Vries, Elisabeth G. E. de Vries, Otto S. Hoekstra, Geke A. P. Hospers, C. Willemien Menke-van der Houven van Oordt

**Affiliations:** 1 Department of Medical Oncology, Cancer Center Amsterdam, Amsterdam UMC, VUmc University Medical Center Amsterdam, de Boelelaan 1117, 1081 HV Amsterdam, The Netherlands; 2 Department of Medical Oncology, University of Groningen, University Medical Center Groningen, Hanzeplein 1, 9713 GZ Groningen, The Netherlands; 3 Department of Epidemiology and Biostatistics, Amsterdam Public Health Institute, Amsterdam UMC, VUmc University Medical Center Amsterdam, De Boelelaan 1105, 1081 HV Groningen, The Netherlands; 4 Department of Nuclear Medicine and Molecular Imaging, University of Groningen, University Medical Center Groningen, Hanzeplein 1, 9713 GZ Groningen, The Netherlands; 5 Department of Radiology and Nuclear Medicine, Cancer Center Amsterdam, Amsterdam UMC, VUmc University Medical Center Amsterdam, De Boelelaan 1117, 1081 HV Amsterdam, The Netherlands; 6 Department of Pathology and Medical Biology, University of Groningen, University Medical Center Groningen, DHanzeplein 1, 9713 GZ Groningen, The Netherlands

**Keywords:** FES PET, FDHT PET, Breast cancer, Oestrogen receptor, Androgen receptor, Interobserver variability

## Abstract

**Purpose:**

Correct identification of tumour receptor status is important for treatment decisions in breast cancer. [^18^F]FES PET and [^18^F]FDHT PET allow non-invasive assessment of the oestrogen (ER) and androgen receptor (AR) status of individual lesions within a patient. Despite standardised analysis techniques, interobserver variability can significantly affect the interpretation of PET results and thus clinical applicability. The purpose of this study was to determine visual and quantitative interobserver variability of [^18^F]FES PET and [^18^F]FDHT PET interpretation in patients with metastatic breast cancer.

**Methods:**

In this prospective, two-centre study, patients with ER-positive metastatic breast cancer underwent both [^18^F]FES and [^18^F]FDHT PET/CT. In total, 120 lesions were identified in 10 patients with either conventional imaging (bone scan or lesions > 1 cm on high-resolution CT, *n* = 69) or only with [^18^F]FES and [^18^F]FDHT PET (*n* = 51). All lesions were scored visually and quantitatively by two independent observers. A visually PET-positive lesion was defined as uptake above background. For quantification, we used standardised uptake values (SUV): SUV_max_, SUV_peak_ and SUV_mean_.

**Results:**

Visual analysis showed an absolute positive and negative interobserver agreement for [^18^F]FES PET of 84% and 83%, respectively (kappa = 0.67, 95% CI 0.48–0.87), and 49% and 74% for [^18^F]FDHT PET, respectively (kappa = 0.23, 95% CI − 0.04–0.49). Intraclass correlation coefficients (ICC) for quantification of SUV_max_, SUV_peak_ and SUV_mean_ were 0.98 (95% CI 0.96–0.98), 0.97 (95% CI 0.96–0.98) and 0.89 (95% CI 0.83–0.92) for [^18^F]FES, and 0.78 (95% CI 0.66–0.85), 0.76 (95% CI 0.63–0.84) and 0.75 (95% CI 0.62–0.84) for [^18^F]FDHT, respectively.

**Conclusion:**

Visual and quantitative evaluation of [^18^F]FES PET showed high interobserver agreement. These results support the use of [^18^F]FES PET in clinical practice. In contrast, visual agreement for [^18^F]FDHT PET was relatively low due to low tumour-background ratios, but quantitative agreement was good. This underscores the relevance of quantitative analysis of [^18^F]FDHT PET in breast cancer.

**Trial registration:**

ClinicalTrials.gov, NCT01988324. Registered 20 November 2013, https://clinicaltrials.gov/ct2/show/NCT01988324?term=FDHT+PET&draw=1&rank=2.

## Introduction

Breast cancer is the most common malignancy in women in the Western world. The majority of breast tumours express the oestrogen receptor (ER), which is the main indicator of potential response to anti-oestrogen therapies [1, 2]. Therefore, it is mandatory to determine ER expression in breast cancer. Recently, the androgen receptor (AR) emerged as a possible target for breast cancer therapy. The AR is present in 70–80% of patients with breast cancer, and AR antagonists are under investigation in clinical trials [3–6].

A tumour biopsy is the gold standard to determine receptor expression. However, this is an invasive procedure, is not always feasible in case of inaccessible tumour sites, and is subject to sampling errors [7]. The 16α-[^18^F]fluoro-17β-oestradiol ([^18^F]FES) and 16β-[^18^F]fluoro-5α-dihydrotestosterone ([^18^F]FDHT) PET/CT have been developed to non-invasively visualise, respectively, the ER and AR status in the tumour lesions within a patient. Previously, it has been shown that [^18^F]FES and [^18^F]FDHT uptake correlate well with ER and AR expression levels in representative breast cancer biopsies [8–10]. As a diagnostic tool, [^18^F]FES PET leads to better diagnostic understanding in 88% and to a change of therapy in 48% of the patients presenting with a clinical dilemma [11]. To predict treatment effects, [^18^F]FES PET can be used to assess residual ER availability during treatment with, e.g. fulvestrant, a selective ER downregulator. Inadequate reduction of the [^18^F]FES PET signal (< 75%) by fulvestrant treatment was associated with early progression [12]. Similarly, in patients with prostate cancer, [^18^F]FDHT PET was used to determine the optimal dose of the AR blocker enzalutamide in a phase 1 trial [13]. Lastly, patients with ER-positive breast cancer and high [^18^F]FDG uptake showed a worse progression free survival if [^18^F]FES uptake was low in comparison to high [^18^F]FES uptake (3 versus 8 months, respectively) [14].

For all these potential applications, reliable, observer-independent identification and quantification of [^18^F]FES and [^18^F]FDHT uptake in tumour lesions is essential for translation to daily clinical practice. Up till now, there are no data on the interobserver variability of [^18^F]FES and [^18^F]FDHT PET in breast cancer. Therefore, the primary objective of this study was to examine interobserver variability in visual and quantitative assessment of [^18^F]FES and [^18^F]FDHT PET. Secondary objectives included the effect of tumour to background ratio (TBR), tracer accumulation, tumour size and the use of different SUV parameters (SUV_max_, SUV_peak_ or SUV_mean_) on interobserver agreement. Also, the added value of quantitative assessment in comparison to visual assessment was examined, and the number of lesions detected on [^18^F]FES and [^18^F]FDHT was compared with those detected on conventional imaging methods (contrast enhanced CT scan and bone scan).

## Materials and methods

### Patient population

This prospective two-centre interobserver variability study was part of a study investigating the correlation between [^18^F]FES and [^18^F]FDHT uptake and ER and AR expression in simultaneously biopsied metastases, of which the results have been published elsewhere [8]. Patients were recruited from September 2014 to August 2015 at the CCA-VUmc University Medical Center Amsterdam and the University Medical Center Groningen in the Netherlands.

Eligibility criteria included metastatic breast cancer and an ER-positive primary tumour, ≥ 1 extrahepatic tumour lesion, ECOG performance status of ≤ 2 and a postmenopausal status or use of LHRH-agonists. Patients were excluded if they had used ER or AR binding drugs during the 6 weeks before study entry, because these ligands compete with tracer binding.

All patients had to give written informed consent before study participation. The study was conducted in compliance with the ethical principles originating in or derived from the Declaration of Helsinki and in compliance with all International Conference on Harmonization Good Clinical Practice guidelines. The local medical ethics committee approved the study (NCT01988324).

### Imaging protocols

[^18^F]FES and [^18^F]FDHT were produced as described previously [15, 16]. On separate days, ≤ 14 days apart, 200 MBq (± 10%) of each tracer was injected. After 60 min (± 5 min), a low-dose CT was performed during tidal breathing for attenuation correction, followed by a whole-body PET scan (skull vertex to mid-thigh, 2 min per bed position). PET/CT scans were made using a Philips Gemini TF-64 PET/CT (Amsterdam) or Siemens 64 slice mCT PET/CT (Groningen). Acquisition and reconstruction protocols used on both scanners were according to the recommendations of the European Association of Nuclear Medicine (EARL) [17].

In addition, a high-resolution, contrast-enhanced CT chest-abdomen and bone scan was performed within 6 weeks of the PET scans for comparison.

### Image analyses

Contrast enhanced CT scans were examined by experienced radiologists and bone scans by experienced nuclear medicine physicians, respectively, masked for the [^18^F]FES and [^18^F]FDHT PET results. Two independent observers from each centre (LM and CV), trained and supervised by two experienced nuclear medicine physicians, performed visual and quantitative analyses. The observers had knowledge of conventional imaging results (contrast enhanced CT and bone scans).

A visually PET-positive lesion was defined as focal uptake above local background incompatible with physiological uptake. Liver metastases were excluded from all analyses in this study because of high physiological [^18^F]FES and [^18^F]FDHT uptake in healthy liver tissue, making reliable identification of metastases difficult. In addition, if visual interpretation of uptake in a (potential) lesion was impossible, e.g. due to overlap with adjacent organs with high physiological tracer, the readers independently reported it as ‘not evaluable’ in the visual ratings, and these were excluded from further analyses. For each patient, the observers made a list that consisted of all lesions already detected on conventional imaging, followed by additional lesions discovered on [^18^F]FES or [^18^F]FDHT PET. An anatomical description of all the lesions was reported in order to match the results. In case a lesion was not reported by one of the two observers, it was scored as not visible for that observer. All visually PET-positive lesions were quantified, as well as PET-negative lesions that were identified on conventional imaging (i.e. lesions on bone scintigraphy and/or high resolution CT > 1 cm).

Each observer manually drew volumes of interest (VOI) on the tumour contours, using PET images for PET-positive lesions and low-dose CT images for PET-negative lesions (lesions only seen on bone scan or high-resolution CT were visually matched on the low-dose CT). Lesions were separately analysed based on visibility on either PET or conventional imaging alone to investigate the influence of visibility on imaging techniques on interobserver agreement.

For every VOI, the standardised uptake values (SUV), i.e. the tracer uptake within a VOI normalised to the injected dose and body weight, were calculated using the software programs accurate (in-house build using IDL, observer 1) and syngo.via version VB10B, Siemens (observer 2). Both programs yielded identical results on test images. Three types of SUV were compared in this study: SUV_max_ (voxel with highest SUV within the VOI), SUV_peak_ (average SUV of a 1 cm^3^ sphere containing the hottest voxels of the VOI) and SUV_mean_ with isocontour 50% of SUV_max_ (average SUV of all voxels with uptake ≥ 50% of SUV_max_).

Based on previous studies, an SUV_max_ [^18^F]FES cut-off ≥ 1.5 was used to define ER-positivity (corresponding with a IHC cut-off of ≥ 1%) and an SUV_max_ [^18^F]FDHT cut-off ≥ 1.9 for AR positivity (corresponding with a IHC cut-off of ≥ 10%) [8, 9].

For [^18^F]FES and [^18^F]FDHT, the SUV_max_ tumour-background ratio (TBR) was defined as the ratio of the SUV_max_ of a tumour lesion and the SUV_mean_ of healthy background tissue. To determine the SUV_mean_ of healthy background tissue, a VOI was drawn on reference tissue in the unaffected contralateral site whenever available or in the unaffected surrounding tissue of the same origin [18].

### Statistical analyses

For visual assessments, agreement was calculated with absolute and relative measures of interobserver agreement. Absolute agreement is the probability that if one observer would score a lesion as visible (positive agreement) or not visible (negative agreement) on the PET scan, the other observer would do the same. It is calculated by the following formulas: positive agreement = 2 × lesions visible to both observers/(2 × lesions visible to both observers + lesions only visible to observer 1 + lesions only visible to observer 2) and negative agreement = 2 × lesions not visible to both observers/(2 × lesions not visible to both observers + lesions only not visible to observer 1 + lesions only not visible to observer 2) [19]. In order to compare results with previous studies, also reliability (relative agreement) was calculated according to Cohen’s kappa, and the results were interpreted as follows: kappa 0.01–0.20 as slight, 0.21–0.40 as fair, 0.41–0.60 as moderate, 0.61–0.80 as substantial and 0.81–1.00 as almost perfect interobserver agreement [20]. To account for potential within-person correlation in visual assessments, a chi-square test was performed to examine whether the percentage visual agreement differed per patient.

For quantitative assessments, parameters are presented as mean ± SD, and reliability was calculated with intraclass correlation coefficients (ICC) using a two-way random effect model with absolute agreement. For the interpretation of the ICCs, the following guideline was used: ≥ 0.90 as excellent, ≥ 0.75 as good, ≥ 0.50 as moderate and < 0.50 as poor [21].

Absolute agreement on quantitative assessments were analysed with Bland-Altman plots (differences between observers showed a normal distribution). For each lesion, it graphically shows the average SUV of observers 1 and 2 on the *x*-axes and on the *y*-axes the difference between observers for each lesion, expressed as percentage of the average SUV value. Percentage differences were used instead of absolute differences to achieve independence of magnitude of differences from magnitude of SUV values, and it facilitates comparisons between the SUV parameters SUV_max_, SUV_mean_ and SUV_peak_, which may show large differences in absolute values.

To investigate the effect of TBRs on interobserver variability, differences between TBRs of [^18^F]FES and [^18^F]FDHT PETs were tested with Wilcoxon matched pairs signed rank tests. In addition, correlations between tracer uptake or tumour size and percentage interobserver differences were determined using the Spearman correlation coefficient (*r*). Finally, linear regression was performed to find the linear function between SUV_max_, SUV_peak_ and SUV_mean_ for [^18^F]FES and [^18^F]FDHT PET, and Cochran’s Q and McNemar tests were used to analyse differences between visibility and quantitative uptake above or below cut-off for SUV_max_, SUV_peak_ and SUV_mean_. *P* value < 0.05 was considered significant. Statistical analyses were generated using the SPSS software (version 22; IBM, SPSS statistics).

## Results

### Patient characteristics

A total of 120 lesions were identified in 10 patients using the different imaging modalities (Table 1). Most lesions were skeletal (66%), followed by lymph node (25%) and visceral metastases (9%). The median number of lesions per patient was 9 (range 2–32).

**Table 1:** Patient characteristics

Characteristic	Number (***n*** = 10)	%
Age in years, mean (range)	67 (48–79)	
Biopsy of primary tumour		
ER+/AR+	10	100
Biopsy of metastases		
ER+/AR+	8	80
ER+/AR-	1	10
ER−/AR−	1	10
Previous treatment lines		
0–1	3	30
2–4	7	70
Visible lesions: total, median per patient (range)	120, 9 (2–32)	
Conventional imaging (CT, bone scan)	69 (54, 40)	58 (45, 33)
Visible on PET alone ([^18^F]FES or [^18^F]FDHT PET)	51 (33, 20)	42 (28, 16)
Total visible on [^18^F]FES PET (observer 1, 2)	64, 69	53, 58
Total visible on [^18^F]FDHT PET (observer 1, 2)	36, 37	30, 31
Location		
Bone (conventional imaging, [^18^F]FES, [^18^F]FDHT PET)	79 (55, 45, 37)	66 (80, 54, 64)
Lymph node (conventional imaging, [^18^F]FES; [^18^F]FDHT PET)	30 (8, 29, 16)	25 (12, 35, 28)
Visceral^a^ (conventional imaging, [^18^F]FES, [^18^F]FDHT PET)	11 (6, 9, 5)	9 (9, 11, 9)

^a^Excluding liver

### Comparison of lesion detection on different imaging modalities

**Fig. 1:**
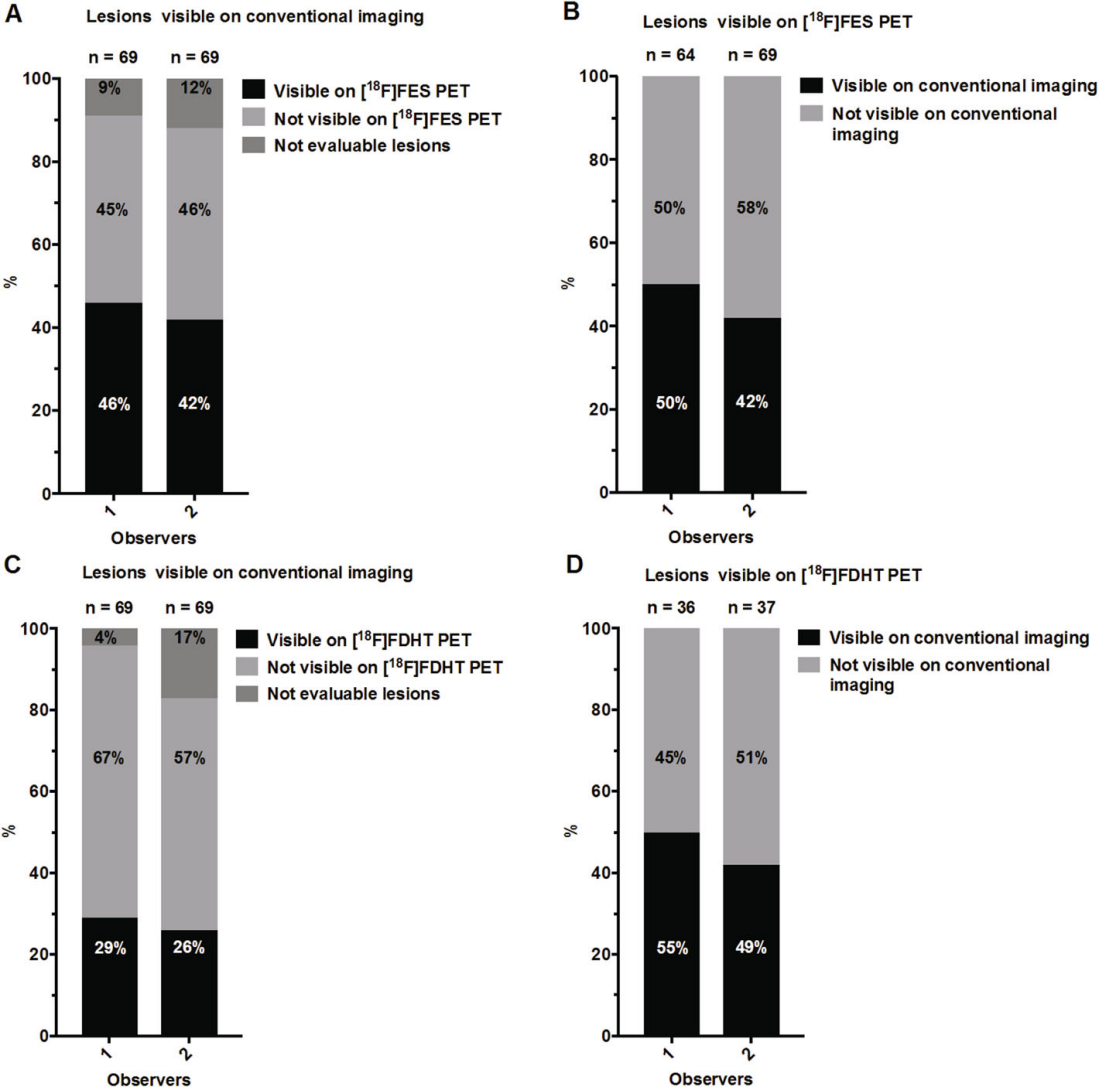
Tumour lesions detected with conventional imaging, [^18^F]FES and [^18^F]FDHT PET

Of the 120 lesions in total (Table 1), most were identified on [^18^F]FES PET (*n* = 64 [53%] and *n* = 69 [58%] by observer 1 and 2, respectively), followed by high-resolution CT (*n* = 54 [45%]), bone scintigraphy (*n* = 40 [33%]) and [^18^F]FDHT PET (*n* = 36 [30%] and *n* = 37 [31%]). Fifty and 42% of the lesions identified on [^18^F]FES PET by observer 1 and 2, respectively, were also detected on high resolution CT or bone scintigraphy (Fig. 1). For [^18^F]FDHT PET, 55% and 49% of the identified lesions were seen with conventional imaging. Conversely, 46 and 42% of the lesions identified on conventional imaging were visible on [^18^F]FES PET by, respectively, observer 1 and 2, and 29% and 26% were seen on [^18^F]FDHT PET. In particular, more lymph node lesions were detected on [^18^F]FES PET and [^18^F]FDHT PET compared to conventional imaging: 97% and 53% versus 27% of all detected lymph node lesions, respectively.

### Visual analysis of [^18^F]FES and [^18^F]FDHT PET images

Out of 120 lesions, a total of 87 and 74 on [^18^F]FES and [^18^F]FDHT PET, respectively, were analysed for visual interobserver agreement. The other lesions were excluded because one or both observers reported these as ‘not evaluable’ due to overlap with adjacent organs with high physiological tracer uptake.

For lesions *visible on conventional imaging*, [^18^F]FES PET readings (Table 2) had substantial positive and negative agreement of 84% (95% CI 72–92%) and 83% (95% CI 70–91%), respectively (kappa = 0.67, 95% CI 0.48–0.87). By including lesions that were only visible on [^18^F]FES PET, the positive agreement improved to 88% (95% CI 80–93%) for *all* lesions scored on [^18^F]FES PET (negative agreement remained the same). [^18^F]FDHT PET showed lower positive agreement of 49% (95% CI 32–65%) for lesions *visible on conventional imaging*, while negative agreement was 74% (95% CI 62–83%) (kappa = 0.23, 95% CI − 0.04–0.49). Positive agreement for *all* lesions scored on [^18^F]FDHT PET was 58% (95% CI 43–71%). By looking at lesions *only visible on PET* and not on conventional imaging, the positive agreement rate was the highest: 91% (95% CI 81–96%) for [^18^F]FES PET and 80% (95% CI 55–93%) for [^18^F]FDHT PET. Visual interobserver agreement was not significantly different between the 10 different patients in this study: *P* = 0.159 for [^18^F]FES PET and *P* = 0.387 for [^18^F]FDHT PET.

**Table 2:** Visual interobserver agreement for lesions visible (A, C) and not visible on conventional imaging (B, D) on [^18^F]FES and [^18^F]FDHT PET, respectively

**A Visual interobserver agreement on [**^**18**^**F]FES PET for lesions visible on conventional imaging**				
	Observer 1			
Observer 2	Visible	Not visible	Not evaluable^a^	Total
Visible	24	3	2	29
Not visible	6	22	4	32
Not evaluable^a^	2	6	0	8
Total	32	31	6	69
**B Visual interobserver agreement on [**^**18**^**F]FES PET for lesions not visible on conventional imaging**				
	Observer 1			
Observer 2	Visible	Not visible	Not evaluable^a^	Total
Visible	26	3	11	40
Not visible	2	1^b^	3	6
Not evaluable^a^	4	1	0	5
Total	32	5	14	51
**C Visual interobserver agreement on [**^**18**^**F]FDHT PET for lesions visible on conventional imaging**				
	Observer 1			
Observer 2	Visible	Not visible	Not evaluable^a^	Total
Visible	9	8	1	18
Not visible	11	27	1	39
Not evaluable^a^	0	11	1	12
Total	20	46	3	69
**D Visual interobserver agreement on [**^**18**^**F]FDHT PET for lesions not visible on conventional imaging**				
	Observer 1			
Observer 2	Visible	Not visible	Not evaluable^a^	Total
Visible	6	2	11	19
Not visible	1	10^c^	6	17
Not evaluable^a^	9	4	2	15
Total	16	16	19	51

^a^Not evaluable lesions due to overlap with adjacent organs with high physiological tracer uptake

^b^Lesions identified on [^18^F]FDHT PET

^c^Lesions identified on [^18^F]FES PET

**Fig. 2:**
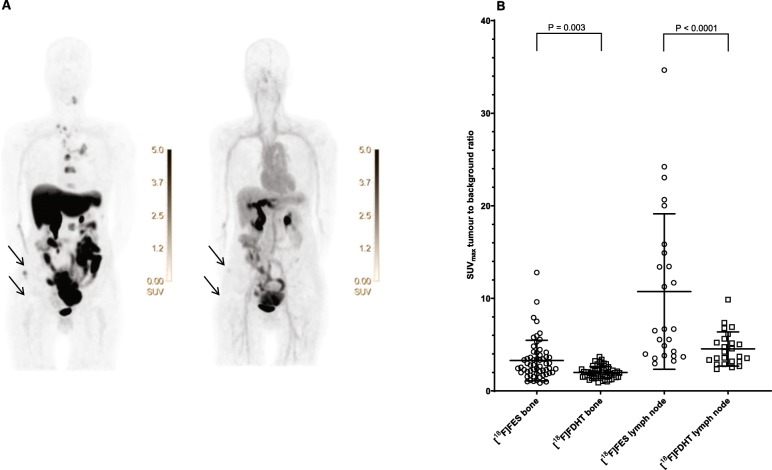
The difference in tumour-background ratio between [^18^F]FES and [^18^F]FDHT PET shown visually (**a**) and quantitatively (mean ± SD) for bone and lymph node lesions (**b**). The arrows in **a** show a bone lesion in the right os ilium visible on [^18^F]FES PET which is only subtly visible on [^18^F]FDHT PET. Note, there is physiological tracer uptake of [^18^F]FES in the liver, gallbladder, intestine, bladder and for [^18^F]FDHT also in the bloodpool

An important aspect in the identification of tumour lesions is how well tracer uptake can be distinguished from background uptake in normal reference tissue. The TBR of [^18^F]FDHT was significantly lower than that of [^18^F]FES (Fig. 2). In bone lesions, the mean TBR of [^18^F]FDHT was 2.0 (± SD 0.6) versus 3.3 (± SD 2.2) for [^18^F]FES (*P* = 0.003). In addition, in lymph node lesions, the mean [^18^F]FDHT TBR was 4.6 (± SD 1.9) compared to 10.7 (± SD 8.4) for [^18^F]FES (*P* < 0.0001).

### Quantitative analyses of [^18^]FES and [^18^F]FDHT PET images

Out of 120 lesions, a total of 94 and 95 were quantified by both observers on [^18^F]FES and [^18^F]FDHT PET, respectively. The other lesions were not quantified by one or both of the observers as a result of overlap with adjacent organs with high physiological tracer uptake, unless there was a clear anatomical substrate on other imaging modalities allowing for reliable VOI definition.

**Fig. 3:**
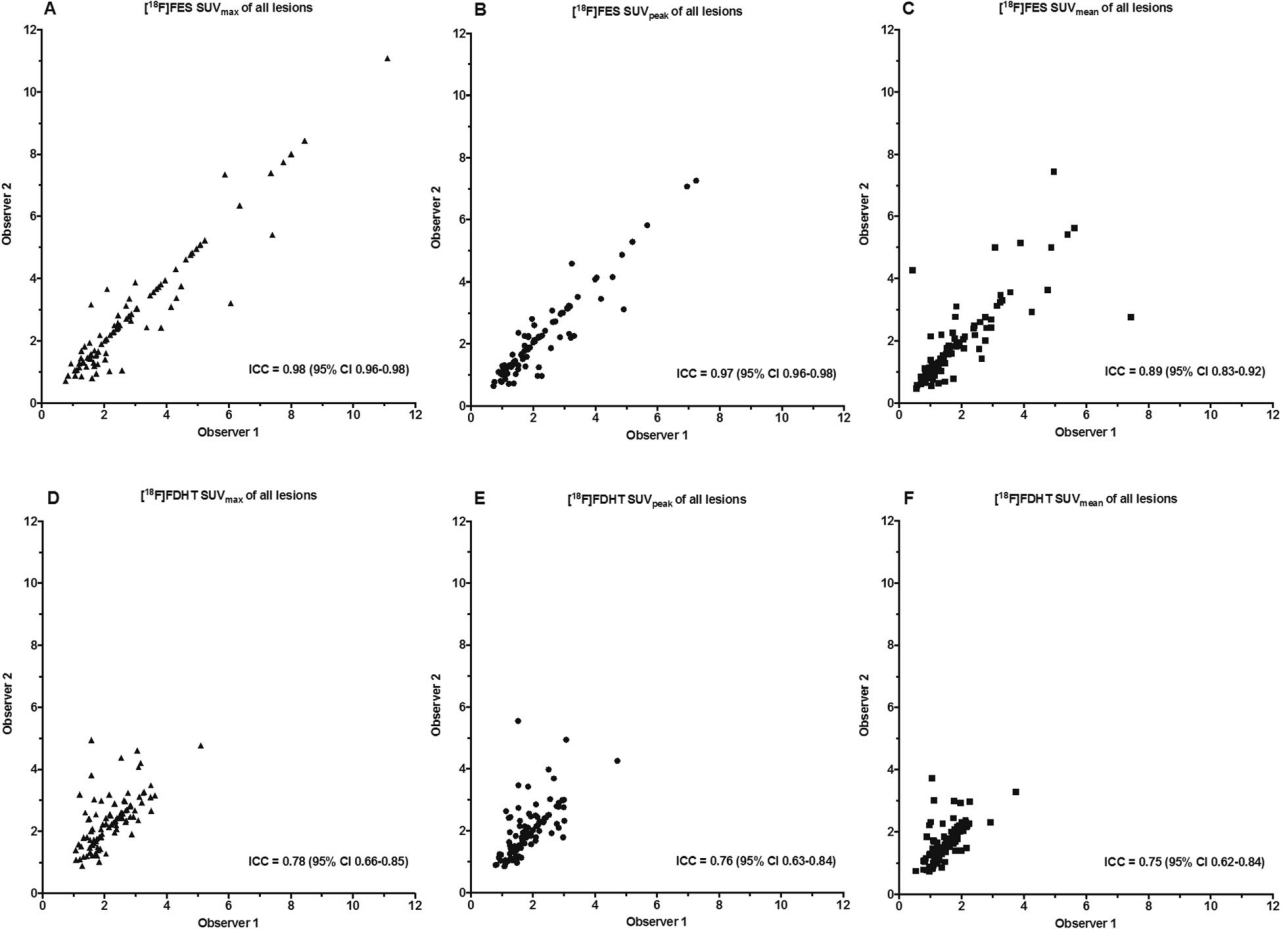
Intraclass correlation coefficients for all quantified tumour lesions on [^18^F]FES (*n* = 94) using SUV_max_, SUV_peak_ and SUV_mean_ (**a**, **b** and **c**) and [^18^F]FDHT PET (*n* = 95) (**d**, **e** and **f**). Note: not quantifiable lesions by one or both of the observers were excluded as a result of overlap with adjacent organs with high physiological tracer uptake

In general, interobserver agreement was excellent for PET quantification (Fig. 3) of *all* lesions combined (i.e. visible on PET or seen on conventional imaging). The ICCs for quantification of SUV_max_, SUV_peak_ and SUV_mean_ on [^18^F]FES PET were 0.98 (95% CI 0.96–0.98), 0.97 (95% CI 0.96–0.98) and 0.89 (95% CI 0.83–0.92). For [^18^F]FDHT PET, the ICCs were lower with 0.78 (95% CI 0.66–0.85), 0.76 (95% CI 0.63–0.84) and 0.75 (95% CI 0.62–0.84), respectively.

**Fig. 4:**
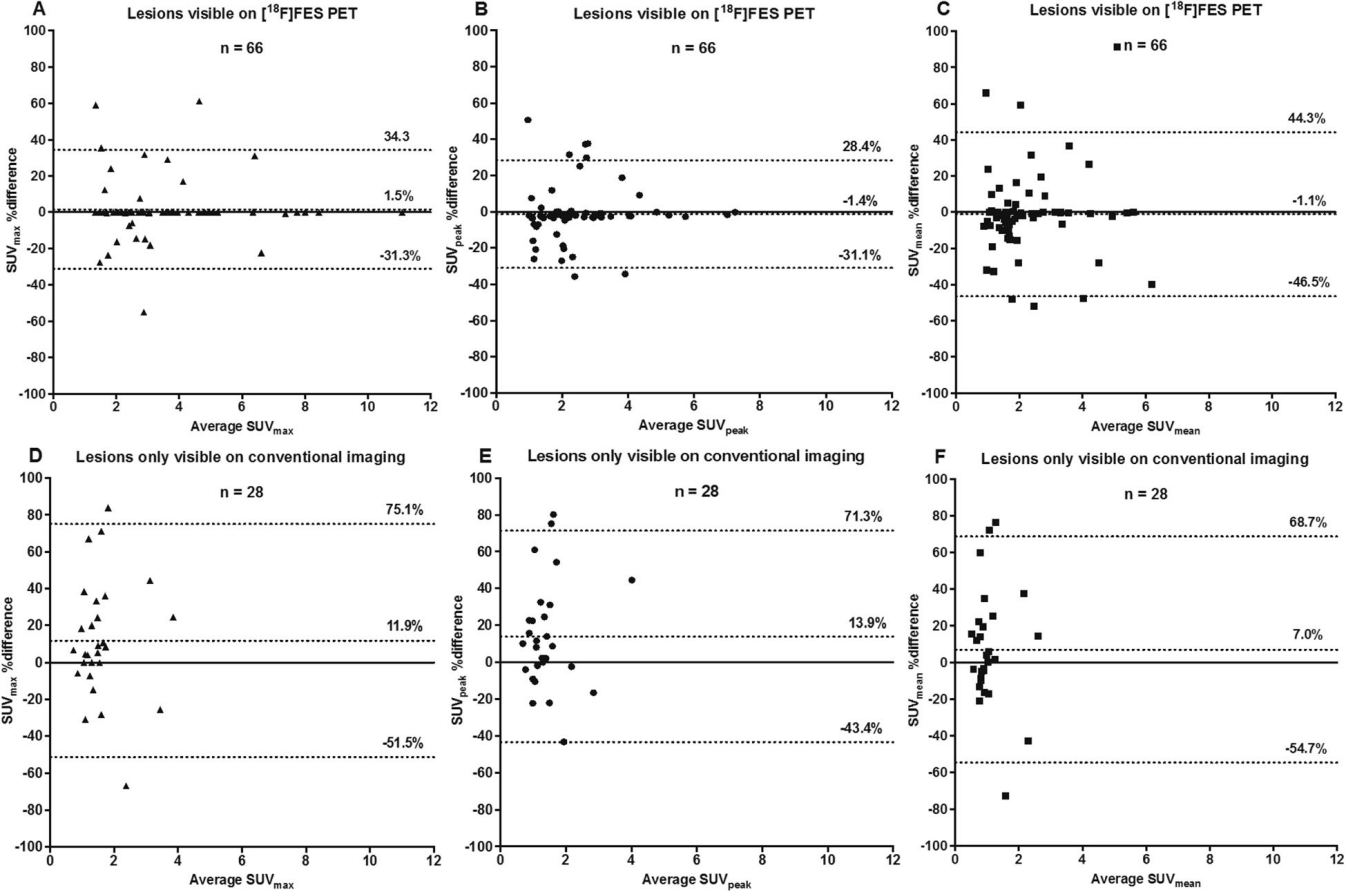
Bland Altman plots showing the % differences in SUV_max_, SUV_peak_ and SUV_mean_ between observers for lesions visible on [^18^F]FES PET (**a**, **b**, **c**) or only visible on conventional imaging (**d**, **e**, **f**). The dashed lines represent the mean difference between observers ± 95% limits of agreement (LOA_95%_)

**Fig. 5:**
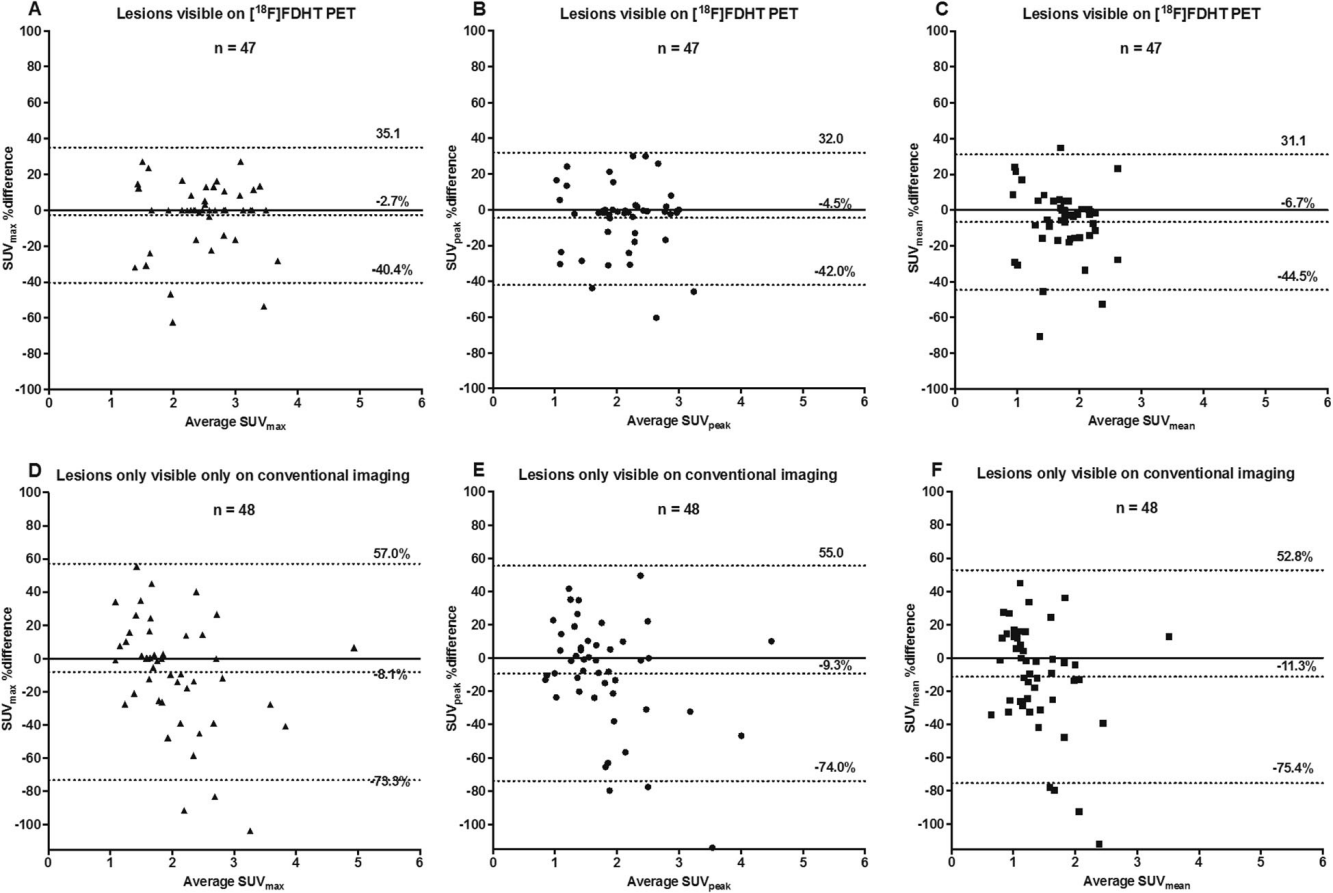
Bland Altman plots showing the % differences in SUV_max_, SUV_peak_ and SUV_mean_ between observers for lesions visible on [^18^F]FDHT PET (**a**, **b**, **c**) or only visible on conventional imaging (**d**, **e**, **f**)

In addition, [^18^F]FES (Fig. 4) and [^18^F]FDHT PET (Fig. 5) quantification was analysed separately with Bland Altman plots for all lesions *visible on PET* or lesions *only visible on conventional imaging* (hence, PET-negative lesions). For [^18^F]FES PET, PET-positive lesions showed excellent quantitative interobserver agreement with mean differences < 2% and 95% limits of agreement (LOA_95%_) being narrower for SUV_max_ (LOA_95%_ − 31.3 to 34.3%) and SUV_peak_ (LOA_95%_ − 31.1 to 28.4%), compared to SUV_mean_ (LOA_95%_ − 46.5 to 44.3%). More differences were shown for PET-negative lesions with mean interobserver differences < 14% and larger LOA_95%_ (within ± 75%), but note that absolute differences between observers were generally low due to a low SUV. Similarly, for [^18^F]FDHT PET, interobserver agreement was better for PET-positive (mean interobserver differences < 7%, LOA_95%_ within ± 45 %) compared to PET-negative lesions (mean interobserver differences < 12%, LOA_95%_ within ± 76%). SUV_max_ and SUV_peak_ showed a better interobserver agreement in comparison to SUV_mean_ for the quantification of lesions visible on [^18^F]FES PET, while on [^18^F]FDHT PET the different SUV parameters were comparable.

Higher levels of tracer accumulation in PET positive lesions were not associated with improved interobserver agreement (for [^18^F]FES PET: Spearman *r* = 0.04, 0.26 and 0.14 for SUV_max_, SUV_peak_ and SUV_mean_, respectively and for [^18^F]FDHT PET: Spearman *r* = 0.00, *r* = 0.03 and *r* = − 0.17, respectively). In addition, there was no correlation between tumour size and interobserver agreement (for [^18^F]FES PET: Spearman *r* = 0.10, *r* = 0.08 and *r* = 0.06, for SUV_max_, SUV_peak_ and SUV_mean_, respectively and for [^18^F]FDHT PET: Spearman *r* = − 0.07, *r* = − 0.16 and *r* = − 0.42, respectively).

### The added value of quantitative assessment in comparison to visual assessment

Based on previous studies, [^18^F]FES and [^18^F]FDHT SUV_max_ cut-off levels of 1.5 and 1.9, respectively, have been identified. There are however limited data on quantitative thresholds and corresponding cut-off values for SUV_peak_ and SUV_mean_. Based on linear regression of all lesions quantified in this study, an SUV_max_ cut-off of 1.5 on [^18^F]FES PET corresponded with an SUV_peak_ of 1.2 and an SUV_mean_ of 1.1 (Supplementary figure S1), and for [^18^F]FDHT PET, an SUV_max_ cut-off of 1.9 corresponded with an SUV_peak_ of 1.6 and an SUV_mean_ of 1.3.

For diagnostic purposes, it is important to identify all receptor positive tumour lesions. Therefore, we compared visual and quantitative tracer uptake above/below cut-off levels (Table 3). In 3% and 1% of the lesions scored visually positive on [^18^F]FES PET by observer 1 and 2 respectively, SUV_max_ was below the threshold of 1.5. For [^18^F]FDHT PET, 14% of the visually positive lesions scored by observer 1 as well as observer 2 had an SUV_max_ below the threshold of 1.9. There were no structural differences between observer 1 and 2. The discrepancies were mostly seen in lesions located in tissue with low background uptake such as skin and lung metastases (Supplementary table S1). Conversely, in 44% and 39% of the lesions scored visually negative on [^18^F]FES PET by observer 1 and 2, respectively, SUV_max_ was ≥ 1.5. Similarly, 31% and 52% of the visually negative lesions had an SUV_max_ ≥ 1.9 on [^18^F]FDHT PET, respectively. However, in most cases (60%), we observed overlap with organs having high physiological tracer accumulation such as the liver and bowel, followed by lesions that were determined to be visually positive at second glance (32%). After correction for these effects, ≤ 4% of the visually negative lesions had a SUV_max_ above cut-off for both tracers.

**Table 3:** Discrepancies between visual and quantitative assessments (above/below cut-off values for receptor positivity) for [^18^F]FES (A) and [^18^F]FDHT PET (B)

**A [**^**18**^**F]FES**	**Observer 1**		**Observer 2**	
	**Visible (*n*= 64)**	**Not visible (*n*= 36)**	**Visible (*n*= 69)**	**Not visible (*n*= 38)**
SUV_max_ ≥ 1.5	62 (97%)	16 (44%)	68 (99%)	15 (39%)
SUV_max_ < 1.5	2 (3%)	20 (56%)	1 (1%)	23 (61%)
SUV_peak_ ≥ 1.2	54 (84%)	19 (53%)	67 (97%)	16 (42%)
SUV_peak_ < 1.2	10 (16%)	17 (47%)	2 (3%)	22 (58%)
SUV_mean_ ≥ 1.1	57 (89%)	8 (22%)	67 (97%)	11 (29%)
SUV_mean_ < 1.1	7 (11%)	28 (78%)	2 (3%)	27 (71%)
**B [**^**18**^**F]FDHT**	**Observer 1**		**Observer 2**	
	**Visible (*n*= 36)**	**Not visible (*n*= 62)**	**Visible (*n*= 37)**	**Not visible (*n*= 56)**
SUV_max_ ≥ 1.9	31 (86%)	19 (31%)	32 (86%)	29 (52%)
SUV_max_ < 1.9	5 (14%)	43 (69%)	5 (14%)	27 (48%)
SUV_peak_ ≥ 1.6	30 (83%)	25 (40%)	33 (89%)	30 (54%)
SUV_peak_ < 1.6	6 (17%)	37 (60%)	4 (11%)	26 (46%)
SUV_mean_ ≥ 1.3	31 (86%)	20 (32%)	33 (89%)	30 (54%)
SUV_mean_ < 1.3	5 (14%)	42 (68%)	4 (11%)	26 (46%)

Not evaluable lesions were excluded as reported in Table 2

Comparing the impact of the different SUV parameters on discrepancies between visual and quantitative assessments showed no significant differences with the only exception that SUV_mean_ showed less visually negative lesions above cut-off on [^18^FES]PET than SUV_max_ or SUV_peak_ for observer 1 (*P* = 0.008 and *P* = 0.001, respectively), but not for observer 2 (*P* = 0.125 and *P* = 0.063, respectively).

## Discussion

Interobserver variability is an important step in the clinical application of diagnostic tools. Here, we showed that both visual and quantitative evaluation were highly reproducible between independent observers evaluating [^18^F]FES PET at separate centres using different scanners and software. Visual positive and negative absolute agreement was > 80%, with a kappa of 0.67. Also, the interobserver reliability of quantitative metrics was excellent for SUV_max_ and SUV_peak_ (ICC of 0.98 and 0.97, respectively) and good for SUV_mean_ (ICC of 0.89). In comparison, staging patients with breast cancer showed similar results for bone scintigraphy (kappa 0.62–0.78) and [^18^F]FDG PET (kappa 0.65 and an ICC of 0.93 for the quantification of [^18^F]FDG uptake) [22–26].

[^18^F]FDHT PET also showed good interobserver reliability for quantitative assessments with ICCs ≥ 0.75. These values are slightly lower than those of [^18^F]FES PET, and this was probably due to the lower lesional [^18^F]FDHT uptake, because quantitative agreement according to Bland Altman analyses were comparable for both tracers. The TBR of [^18^F]FDHT was considerably lower compared to [^18^F]FES. This probably explains the higher variability in visual interpretation (kappa = 0.23), mainly caused by a low visual positive agreement (49%) in lesions already identified by conventional imaging modalities, while positive agreement in lesions not identified by conventional imaging was much higher (80%), as well as negative visual agreement between observers (74%). An important impeding factor was the significantly lower TBR of [^18^F]FDHT in bone and lymph node lesions compared to [^18^F]FES PET. The TBR of [^18^F]FDHT in the current study (2.0 for bone and 4.6 for lymph nodes) was also lower than in prostate cancer metastases (3.3 for bone and 5.7 for soft tissue metastases) with an SUV_max_ three times higher in prostate cancer (7.1–9.1 versus 2.0 in the present breast cancer study) [27, 28]. This suggests that higher AR expression likely results in better interobserver reliability.

Our study had some limitations. There were only a limited number of patients included in this study. However, receptor expression between lesions within a single patient can be heterogeneous [29], which was confirmed in the present study resulting in the coverage of a large range of data in 120 lesions [8]. In addition, we showed there was no within-patient correlation in visual assessments. A second limitation is a substantial number of ‘not evaluable’ lesions, due to overlap with adjacent organs with high physiological background. The decision for evaluability was left to each observer individually, which may have contributed to the low agreement (≤ 6%) on these ‘not evaluable lesions’. For future studies, we recommend that all lesions with physiological background overlap from the liver, gallbladder, intestine, bladder and for [^18^F]FDHT also from bloodpool are regarded as not evaluable. A third limitation is the lack of robust [^18^F]FES and [^18^F]FDHT thresholds for test positivity. We used an SUV_max_ cut-off of 1.5 for [^18^F]FES and 1.9 for [^18^F]FDHT PET based on previous data corresponding with ER and AR positivity in biopsies and so far showing the best predictive value for response to endocrine therapy [8, 9, 30, 31]. Some studies suggested an SUV_max_ cut-off of 2.0 for [^18^F]FES PET, taking into account the background [^18^F]FES uptake in normal tissues which can exceed the cut-off of 1.5 [29–31]. Tissue specific cut-off values may indeed be more appropriate as there are responders to endocrine therapy with a tumour SUV_max_ < 2.0. In the current study, up to 20% of the visually positive lesions had an SUV_max_ < 2.0, while < 3% had an SUV_max_ < 1.5 (Supplementary table S2).

For diagnostic purposes, simple visual assessment of [^18^F]FES uptake may suffice to determine the receptor status of a tumour lesion (agreement was high between visual assessment and the applied SUV_max_ cut-off value of 1.5 for ER-positivity). True discrepancies between visibility and corresponding uptake above or below cut-off were low (< 4%), making quantification of visually negative lesions not only cumbersome, but also unnecessary. Also, quantification of lesions without visual [^18^F]FES uptake leads to higher interobserver variability due to differences in VOI definition. However, quantification remains a helpful tool for nuclear medicine physicians in ‘equivocal [^18^F]FES lesions’. In addition, quantification is useful to measure receptor availability over time for the evaluation of treatment effects. In contrast, quantification of [^18^F]FDHT uptake is still required in future breast cancer studies, as we have shown relatively low visual agreement.

The role of [^18^F]FES and [^18^F]FDHT PET in addition to conventional imaging modalities needs to be defined further. It has to be taken into account that besides partial volume effects and constraints due to background tracer uptake limiting their detection, receptor expression can be heterogeneous and variable during the course of the disease [11, 32]. In addition, treatment may induce changes in receptor expression, but also eradicated tumour cells can leave a visible lesion on conventional imaging (e.g. sclerotic bone lesions), in absence of viable tumour cells. In the current study with heavily pretreated patients, 42–46% and 26–29% of the lesions identified by conventional imaging were detected on [^18^F]FES and [^18^F]FDHT PET, respectively. Vice versa, only approximately 50% of the lesions observed on [^18^F]FES PET and [^18^F]FDHT PET were identified by conventional imaging.

Therefore, a potential role for [^18^F]FES PET may be in staging of early ER-positive breast cancer as an addition to existing imaging techniques. Standard staging with [^18^F]FDG PET can miss low-intermediate grade ER-positive lesions due to their low metabolic activity [33]. We are currently investigating [^18^F]FES PET in staging patients with low grade, ER-positive locally advanced or recurrent breast cancer versus [^18^F]FDG PET (NCT03726931), and in metastatic breast cancer versus addition to conventional diagnostics (NCT01957332). The *non-invasive* visualisation of receptor status in metastatic lesions with PET offers a number potential clinical advantages. For example, in case conventional diagnostics cannot establish a final diagnosis of suspected metastatic breast cancer lesions (e.g. as a result of inaccessible biopsy sites or repeated biopsy sampling errors). Also, PET imaging may help to determine the hormone receptor status of *different tumour sites within a patient* and guide treatment decisions, for instance, to decide on the origin of a metastatic lesion in case of multiple primary tumours or to determine whether receptor conversion occurred in metastases from a single primary tumour [11]. If validated, this may help with multimodality treatment strategies for heterogeneous tumour sites of breast cancer, such as endocrine therapy for [^18^F]FES positive lesions combined with a local modality such as radiotherapy for concurrent [^18^F]FES negative lesions [34].

## Conclusion

In conclusion, our findings demonstrate that visual and quantitative evaluation of [^18^F]FES PET has a high interobserver concordance and support the use in clinical practice. Although [^18^F]FDHT PET showed relatively low visual agreement, presumably a result of the low AR expression and consequently low TBR in patients with breast cancer, there was good quantitative agreement between observers, acceptable for further [^18^F]FDHT PET imaging studies in breast cancer.

## Data Availability

The datasets used and/or analysed during the current study are available from the corresponding author on reasonable request.

## Abbreviations

ER: Oestrogen receptor
AR: Androgen receptor
[^18^F]FES: 16α-[^18^F]fluoro-17β-oestradiol
[^18^F]FDHT: 16β-[^18^F]fluoro-5α-dihydrotestosterone,
VOI: Volume of interest
SUV: Standardised uptake value
TBR: Tumour-background ratio
ICC: Intraclass correlation coefficient
LOA_95%_: 95% limits of agreement
